# MacArthur on Habitual Costiveness

**Published:** 1850-09

**Authors:** Erial MacArthur

**Affiliations:** Chicago, Ill.


### ARTICLE IX.

*Nitro-Muriatic Mixture in Habitual Costiveness.*—By ERIAL MACARTHUR, M. D., Chicago, Ill.

Having been called upon to prescribe for an infant daughter of Wm. McDearbon, on Clark St., for continued costiveness, and finding that all the usual remedies afforded only present relief, and sometimes not even that, until cathartics of different kinds had been given for several days, I determined to see what efficacy there might be in the Nitro-Muriatic Acid Mixture in a case of this kind.

Feb. 17th, 1850. I made the following prescription, to wit: ℞. Nitric and Muriatic Acid, aa. ʒj.; Rain Water, ℥j. Mix. Apply with a sponge or soft linen a little of it morning and evening over the region of the liver and right iliac region, until the alvine evacuations are produced plentifully.

This effect was produced in two or three days after the first application, and the patient has not been afflicted with costiveness to any extent since, and although feeble up to that time has enjoyed excellent health since. It may be well to state that the patient was at this time one year old, and had been afflicted with this continued costiveness from birth. The mother informed me from time to time, that she was obliged to give the babe physic almost every week, and sometimes twice or thrice a-week.

I had prescribed for the patient many times during the year for this difficulty, but had never been able to produce anything more than temporary relief.

I have occasionally made the same prescription for adult patients of both sexes, for habitual costiveness, where occasioned by a torpid and inactive state of the liver; and almost invariably with entire success.

I might add that I have avoided the use of this mixture, when patients appeared to have local inflammation of any tissue or organ of the system.

